# Comprehensive Evaluation of Vocal Outcomes and Quality of Life after Total Laryngectomy and Voice Restoration with J-Flap and Tracheoesophageal Puncture

**DOI:** 10.3390/cancers14030544

**Published:** 2022-01-21

**Authors:** Chung-Kan Tsao, Filippo Marchi, Chung-Jan Kang, Claudio Sampieri, Yi-An Lu, Shiang-Fu Huang, Yu-Ting Chen, Giorgio Giordano, Giorgio Peretti, Giampiero Parrinello, Andrea Iandelli, Tuan-Jen Fang

**Affiliations:** 1 Department of Plastic and Reconstructive Surgery, Chang Gung Memorial Hospital, Chang Gung Medical College, Chang Gung University, Taipei 333, Taiwan; nightman@cgmh.org.tw (C.-K.T.); filippo.marchi@unige.it (F.M.); kevin955282@gmail.com (Y.-T.C.); 2 Center for Tissue Engineering, Chang Gung Memorial Hospital, Taoyuan 333, Taiwan; 3 Department of Otolaryngology, IRCCS Ospedale Policlinico San Martino, 16132 Genoa, Italy; s4584120@studenti.unige.it (C.S.); s3627659@unige.studenti.it (G.G.); giorgio.peretti@unige.it (G.P.); giampiero.parrinello@gmail.com (G.P.); 4 Department of Surgical Sciences and Integrated Diagnostics (DISC), University of Genoa, 16132 Genoa, Italy; 5 Department of Otolaryngology, Chang Gung Memorial Hospital, Chang Gung Medical College, Chang Gung University, Taipei 333, Taiwan; keny@adm.cgmh.org.tw (C.-J.K.); b9402009@cgmh.org.tw (Y.-A.L.); bigmac@adm.cgmh.org.tw (S.-F.H.)

**Keywords:** laryngeal cancer, total laryngectomy, voice rehabilitation, free flap, microsurgery, quality of life

## Abstract

**Simple Summary:**

Laryngopharyngectomy is still the treatment of choice in locally advanced pharyngolaryngeal tumors not eligible for organ preservation protocols. Loss of speech capacity has been reported as one of the factors that most affect the patient-reported quality of life. Thus, the reconstructive goals are restoring the pharynx and possibly the voice in such a scenario. For decades, tracheoesophageal puncture (TEP) has allowed proper voice rehabilitation; however, TEP has a non-neglectable financial expenditure and complication rate. Therefore, we recently reported a novel flap design and surgical technique that shares the same principles of TEP, without the need to change any device over time, named J-flap. This study aimed to analyze both techniques’ subjective and objective vocal outcomes and their impact on overall and voice-related quality of life.

**Abstract:**

Background: Tracheoesophageal puncture with a voice prosthesis is the gold standard for speech rehabilitation in patients that receive a laryngopharyngectomy. However, a novel surgical technique, using a tubularized anterolateral tight flap, named “J-flap,” has been demonstrated to produce adequate voice restoration. We aimed to compare the outcomes and the quality of life of patients who underwent voice rehabilitation with both techniques. Methods: We enrolled patients that underwent laryngopharyngectomy and voice restoration surgery. The control group received a tracheoesophageal puncture with a voice prosthesis, while the study group received J-flap reconstruction. A total of 20 patients received voice prosthesis rehabilitation, while 18 received J-flap reconstruction. Speech and vocal outcomes and quality of life metrics were collected. Results: The objective phonatory performances and the acoustic voice analysis did not outline a significant difference. Speech pathologists judged the consonant pronunciation in the J-flap group as less accurate (*p* < 0.001). The voice handicap index revealed a moderate impairment for the J-flap group (*p* < 0.001). Quality of life scores were higher for the voice prosthesis group. Conclusion: Voice prostheses and J-flaps share similar objective phonatory outcomes. Quality of life was more impaired in the J-flap group. In our view, these two techniques possess complementary characteristics in clinical practice, taking into account health care system regulations and patients’ social background.

## 1. Introduction

Laryngopharyngectomy (LP) is the treatment of choice in locally advanced tumors affecting the pharyngolaryngeal junction not eligible for organ preservation protocols, or as a salvage strategy of a previously irradiated larynx and/or hypopharynx [1]. Even if LP is a sound oncological treatment, its functional consequences often lead to a dismal quality of life (QoL), mainly due to the loss of the ability to speak [2]. Therefore, in recent decades, multiple efforts have been directed towards patients’ vocal rehabilitation. To date, after LP or total laryngectomy (TL), the voice can be restored mainly with three methods: by esophageal speech (ES), by using external voice devices such as an electrolarynx (EL) or, finally, by a tracheoesophageal puncture (TEP) with the placement of artificial valves (voice prostheses) [3]. However, since the introduction of the first voice prosthesis (VP) by Singer and Blom in 1980 and its more modern versions, which have dramatically improved the QoL after laryngectomies, no further safe and effective solution has been developed to rehabilitate laryngectomy patients [4].

As free flaps represent the standard of care for pharyngeal reconstruction [5], an approach that exploits both the need for pharyngeal restoration and phonatory rehabilitation in a single-stage procedure, without requiring any additional device, might be an appealing strategy in this field.

Autologous free flaps for laryngopharyngeal reconstruction allow single-stage synchronous reconstruction of both the esophagus and voice, with promising phonation success [6,7,8,9,10]. These advancements of voice tube reconstruction were based on various concepts and designs of tracheoesophageal shunts. The authors recently published their voice restoration technique using a J-shaped anterolateral thigh (ALT) free flap, shaped into a phonatory tube, called “J-flap”. The functional results demonstrated the J tube could provide a reliable and satisfactory voice rehabilitation method [11]. This study aimed to compare the functional outcomes and the QoL of patients who underwent voice rehabilitation with the J-flap technique and the gold standard vocal rehabilitation method of TEP with voice prostheses.

## 2. Materials and Methods

This study was conducted in accordance with the Declaration of Helsinki and was approved by the local ethics committees (CER Liguria: 230/2019, and IRB Taiwan: 202000478B0).

We conducted a retrospective, multicentric study at two independent hospitals: the Department of Otolaryngology—Head and Neck Surgery of IRCCS Ospedale Policlinico San Martino, Genoa, Italy, and the Department of Otolaryngology—Head and Neck Surgery and the Department of Reconstructive Surgery of Linkou Chang Gung Memorial Hospital, Taoyuan, Taiwan. Data were collected from October 2017 to September 2019 in Genoa, and from August 2017 to September 2019 in Taiwan, on a cohort of consecutive patients affected by laryngeal or hypopharyngeal squamous cell carcinoma. The inclusion criteria were as follows: age over 18 years old, a biopsy-proven squamous cell carcinoma (SCC) of the larynx/hypopharynx, at least six months of post-treatment follow-up, patients treated by ablative surgery with curative intent and voice restoration surgery. In addition, we excluded patients with synchronous head and neck squamous cell carcinoma and the presence of distant metastases.

All patients had been submitted to larynx-ablating surgery after multidisciplinary tumor board (MDTB) discussion and preoperative counseling between head and neck surgeons, and radiation and medical oncologists. Postoperative radiotherapy (RT) or chemo-radiotherapy (CRT) administration was discussed by the MDTB and offered to patients according to the National Comprehensive Cancer Network (NCCN) guidelines [12]. Tumors were classified according to the 8th Edition of the AJCC UICC TNM staging system [13]. All patients underwent TL or LP with partial/total sacrifice of the surrounding pharynx. Selective (SNDs) or modified radical neck dissections (MRNDs) were performed in adherence with NCCN guidelines [12]. Voice restoration surgery was provided for the entire cohort. The subgroup treated at San Martino hospital received a primary or secondary tracheoesophageal puncture (TEP), whereas the Chang Gung Memorial hospital patients were treated with J-flap phonatory tube reconstruction. All data concerning comorbidities, demographics, preoperative staging, type of surgery, surgical outcomes, histopathology and follow-up were collected in a single dedicated database. In addition, speech outcomes and QoL metrics were collected prospectively with a shared protocol between the two hospitals. All the tests were performed in Italian for the San Martino hospital cohort, and Mandarin for the Chang Gung Memorial hospital patients, at least six months after treatment.

### 2.1. Surgical Technique

#### 2.1.1. Tracheoesophageal Puncture

The TEP rationale is to create a communication between the trachea and the esophagus so that the air contained in the lungs can be pushed into the neopharynx by passing through the surgically created fistula. The resonation obtained from the mucosa vibration results in the sound being articulated through the mouth to produce speech. A unidirectional silicon valve is placed into the fistula to impede the oral diet and secretions from passing through the fistula. The TEP puncture and the first insertion of the valve are performed under general anesthesia. The procedure can be performed in the context of a primary laryngeal ablation or postponed generally after several months, considering the patient’s willingness and tissue healing after the primary intervention. In this study, all the prostheses applied were Provox Vega (Atos Medical, Malmö, Sweden) 22.5 Fr with different sizes according to the fistula’s depth (See Figure S1 and Video S1).

#### 2.1.2. J-Flap Phonatory Tube

**Figure 1:**
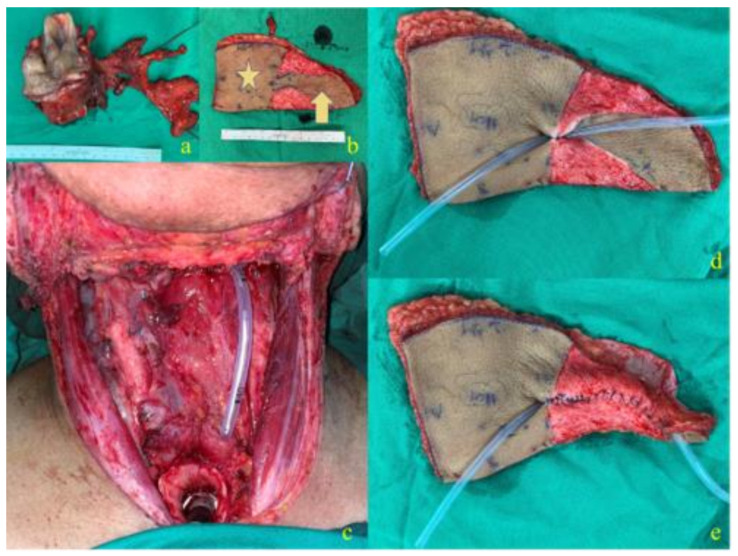
Demonstration of the design of the J-flap: (**a**) the excised tumor specimen; (**b**) proximal part of the J-flap (star), and distal part of the J-flap (arrow); (**c**) the tracheoesophageal defect; (**d**,**e**) creation of the phonation tube.

**Figure 2:**
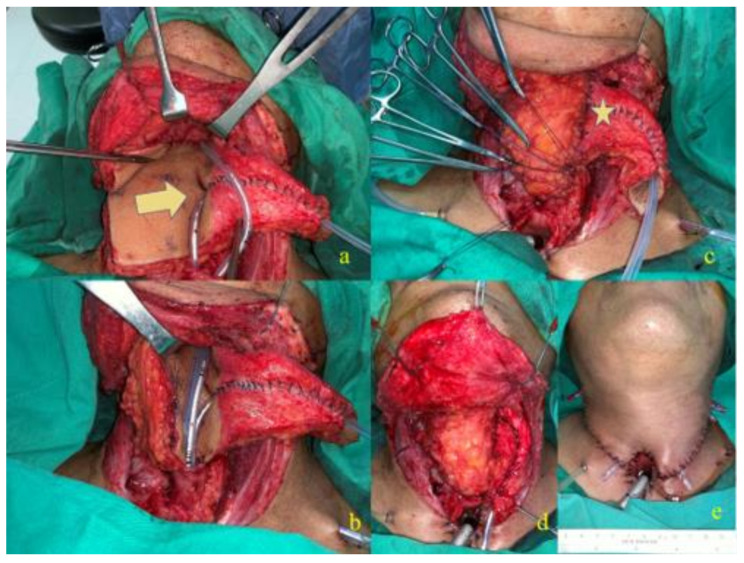
Demonstration of the inset of the J-flap: (**a**) inset of the proximal esophagus, and internal orifice of the phonation tube (arrow); (**b**) inset of the distal esophagus; (**c**) inset of the esophageal tube, and proximal portion of the phonation tube (star); (**d**) inset of the distal orifice of the phonation tube to the tracheostoma; (**e**) inset of the cervical flap, and completed reconstruction.

Figure 1 and Figure 2 demonstrate the design and inset of the J-flap to reconstruct the esophageal defect and create a phonation tube. The J-flap is made with an anterolateral thigh free flap. It is composed of a proximal part (a trapezoidal section) (Figure 1b, star), used for pharyngeal/esophageal reconstruction, and a distal portion (a dome-shaped section) (Figure 1b, arrow), used to create the phonatory tube. The distal part of the flap is tabularized, sutured on itself, and a catheter is inserted to maintain the patency of the tract during healing (Figure 1d,e). The phonatory tube forms an angle >90° to the proximal part of the flap to prevent food regurgitation or aspiration (Figure 2c, star). The distal portion of the tube is sutured to the lateral wall (either left or right) of the stoma (Figure 2d,e); the proximal part opens up into the neopharyngeal lumen, and its orifice has an elliptical shape (Figure 2a, arrow) and a smaller diameter compared to the distal orifice to prevent aspiration. The voice is produced by the occlusion of the tracheal stoma, meaning the air from the trachea is diverted to the esophagus. As for TEP, the air causes phonation tube or mucosa vibration, and the sound articulation with the mouth allows the patient to produce the voice. For the detailed surgical technique, please refer to the index paper [14] (See Figure S2 and Video S2).

### 2.2. Speech and Voice Evaluation

#### 2.2.1. Speech Intelligibility

Speech intelligibility was rated using the National Technical Institute for the Deaf (NTID) rating scale, a 5-point Likert scale with the best rated as 5 and the worst rated as 1. The test was first performed by recording the speech when the patient read a standard passage. Then, an experienced speech pathologist rated the speech samples blinded to the medical information of the speaker.

#### 2.2.2. Speech Accuracy

The speech accuracy was evaluated by reading a standard word set containing 74 phonetically balanced words. Readers were recorded, and the samples were assessed by 2 experienced speech pathologists who judged the number of correct words, vowels and consonants, still blinded to the medical information of the speaker. The Italian patients spoke Italian and were evaluated by Italian SLPs, whereas the Taiwanese patients spoke Mandarin and were evaluated by Taiwanese SLPs. The NTID rating scale and speech accuracy have been proven to have good intra- and inter-rater reliability and validity in previous studies [15,16].

#### 2.2.3. Voice Handicap Index

The VHI is a tool to measure the patient’s voice handicap and its related quality of life [17,18]. It consists of 30 questions from 3 items to evaluate the functional, physical and emotional aspects of the life quality. To sum up, these three parts create a final score. A score of 0 to 30 correlates with minimal impairment, while a score of 31 to 60 reflects a moderate handicap, and a VHI total score from 61 to 120 is considered to reflect a severe handicap [19].

#### 2.2.4. Subjective Assessment of Dysphonia (GIRBAS)

The subjective voice assessment of dysphonia was performed by speech language pathologists using the GIRBAS scale. GIRBAS is an acronym that stands for grade of dysphonia (G), instability of the voice (I), roughness (R), breathiness (B), asthenia (A) and strain (S). Each of these parameters is scaled from 0 (normal) to 3 (most impaired). The global dysphonia grade is then rated as grade 1 (normal voice), grade 2 (mild dysphonia), grade 3 (moderate dysphonia), grade 4 (severe dysphonia) or grade 5 (aphonic) [20].

#### 2.2.5. Acoustic Voice Analysis

Laboratory acoustic voice analysis was performed by using the CSL4500B 5.05 software (Kay-PENTAX, Montvale, NJ, USA). We asked the patients to produce a sustained vowel, namely, /a/, at a conversational pitch and loudness. The instrument analyzed the voice produced to calculate the fundamental frequency (F0), jitter, shimmer and harmonic-to-noise ratio (HNR) [11].

#### 2.2.6. Maximum Phonation Time

Maximum phonation time (MPT) was assessed by timing the patients who were asked to produce the vowel /a/ for as long as they could after a deep inhalation.

The S/Z ratio is the duration of the longest sustained /s/ to /z/, which characterizes the ability of mucosal vibration; the ideal value should be close to 1.

### 2.3. Quality of Life Evaluation: The University of Washington Quality of Life Questionnaire

The University of Washington quality of life questionnaire (UW-QoL) was first reported by Rogers et al. [21] and is specific for head and neck patients. The test comprises 12 single questions, each with 3 to 6 options for choice that are scaled from 0 (worst dysfunction) to 100 (normality) according to the patient’s disturbance or well-being. The last version of the UW-QoL (version four) was adopted, and the domains evaluated were pain, appearance, activity, recreation, swallowing, chewing, speech, shoulder, taste, saliva, mood and anxiety [22]. Scores for these are calculated independently and range from 0 (as the worst) to 100 (the best). The mean between the 12 items results in a composite score which is usually used as the QoL indicator. As outlined by Rogers et al. [21], this outcome can also be reported using two subscales of physical (chewing, swallowing, speech, taste, saliva and appearance) and social-emotional (anxiety, mood, pain, activity, recreation and shoulder) function: each subscale is based on the average of six items that are used to derive it. Finally, the questionnaire also includes three questions related to global QoL, which have scores ranging from 0 to 100 as well.

### 2.4. Statistical Analysis

The Bioinfokit [23] toolkit and SPSS program (SPSS, v. 23.0, IBM, Armonk, NY, USA) were used for statistical analysis. The differences between patients’ characteristics, voice and QoL metrics in the two groups were compared by Mann–Whitney’s U test, while Welch’s *t*-test was used for continuous variables and the chi-squared test for categorical variables. Descriptive statistical values were expressed as the mean with the standard deviation. A P-value less than 0.05 was considered to be statistically significant.

## 3. Results

A total of 38 patients were enrolled in the study: 20 (52.6%) from San Martino hospital and 18 (47.4%) from Chang Gung Memorial Hospital. Patients’ characteristics, tumor site, staging, surgery, preoperative and postoperative radiotherapy status and voice restoration surgery are presented in Table 1. The average age of the patients in the TEP group was 61.70 ± 7.71 years, while the average age of the patients in the J-flap group was 58.33 ± 9.39 years. All patients in both groups were males. The weight and height difference between the TEP and J-flap groups was statistically different (respectively, 72.00 ± 8.68 kg vs. 62.82 ± 8.63 kg; *p* = 0.002, and 1.72 ± 0.04 m vs. 1.65 ± 0.05 m; *p* < 0.001), while, broadly, BMI showed no statistically significant difference (24.05 ± 2.53 vs. 22.96 ± 2.82; *p* = 0.2). The locations of the primary tumor were significantly different between the two groups, with a prevalence of the larynx in the TEP group, and the pharynx in the J-flap group (*p* < 0.001). There was a statistically significant (*p* < 0.001) difference in radiotherapy administration both in the preoperative and postoperative settings between the two groups: in the TEP group, 4 (20%) patients underwent preoperative radiotherapy, 15 (75%) underwent postoperative radiotherapy and 1 (5%) did not undergo radiotherapy, while in the J-flap cohort, 15 (83.3%) patients received preoperative radiotherapy and 3 (16.7%) received postoperative radiotherapy.

**Figure 3:**
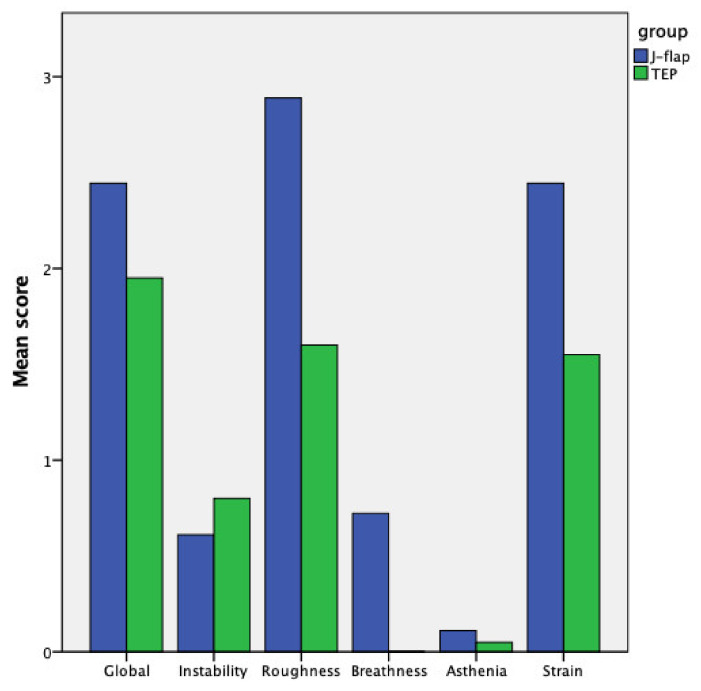
Dysphonia assessment—GIRBAS score.

In regard to the speech pathologists’ judgment using the GIRBAS scale, the J-flap group performed significantly worse in all fields except for instability (Figure 3). Globally, the speech was judged less intelligible according to the NTDI scale for the J-flap group compared to the TEP group (3.50 ± 0.63 vs. 4.25 ± 1.11; *p* = 0.005). Finally, some statistically significant differences arose in the speech accuracy test, where the J-flap group was judged to be less accurate in the correct pronunciation of consonants (65.77% ± 11.02 vs. 86.02% ± 20.79; *p* < 0.001). Speech pathologists’ evaluations are reported in Table 2.

The phonatory performances in terms of the MPT and S/Z ratio were comparable in both groups. Even the acoustic voice analysis did not outline any statistically significant differences. All the acoustic and phonatory parameters are reported in Table 3.

**Figure 4:**
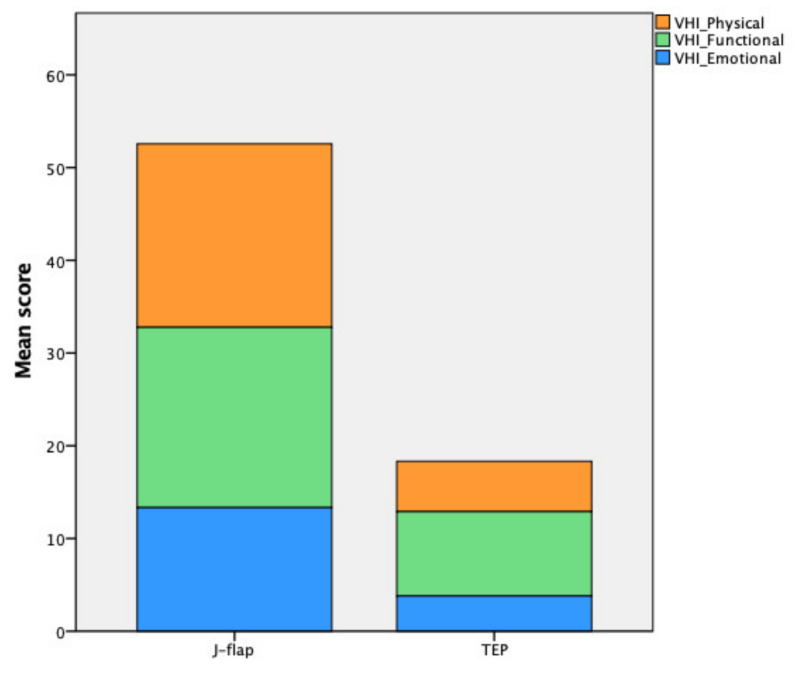
Voice handicap index scores.

The subjective evaluation provided by patients concerning their voice through the VHI revealed a comprehensively significant difference between the two cohorts (*p* < 0.001), with a moderate impairment for the J-flap group (52.56 ± 26.78) and a minimal impairment for the TEP group (18.32 ± 11.62). The results of the VHI test with each domain score are reported in Table 4 and in Figure 4.

**Figure 5:**
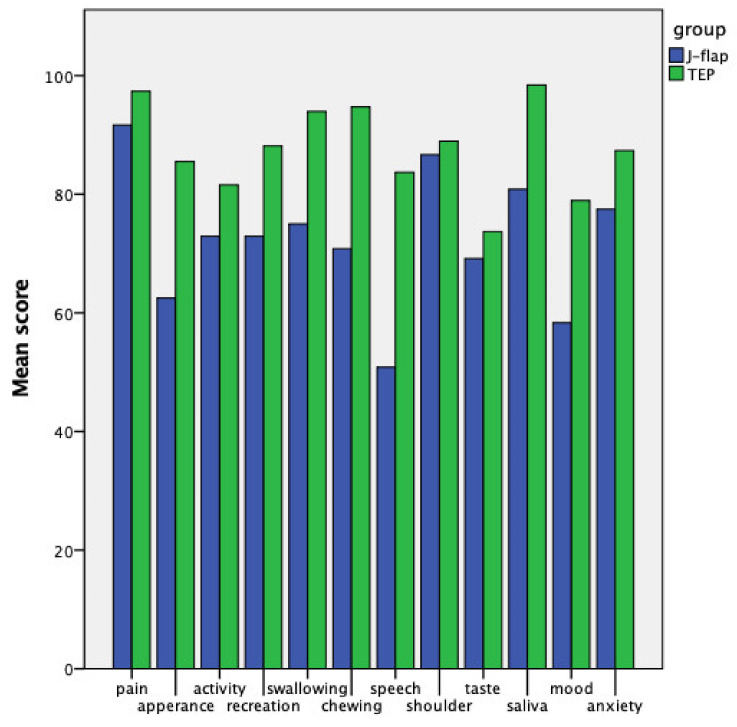
The University of Washington quality of life questionnaire.

Finally, concerning the assessment of the QoL through the UW-QoL, the scores were higher for the TEP group in each of the 13 entries (Figure 5), with a complex composite score significantly more favorable for the TEP group (*p* = 0.016). Still, when analyzed in subgroups, statistical significance was reached only for the physical function subdomain (*p* = 0.009). The UW-QoL results are reported in Table 5.

## 4. Discussion

TLP and TL used to carry a tremendous impact on patients’ lives, but, nowadays, voice restoration techniques have facilitated reintroduction of patients into their social background, significantly reducing the impact of their mutilation [24]. These techniques have evolved from ES and EL to more efficient methods such as TEP. The latter has been demonstrated to allow better voice performances as it recreates the normal air route from the lungs through the pharynx to the mouth, where the voice is articulated: this allows a more prolonged, fluent and easy-to-learn elocution compared to ES, and a more natural sound, less stigmatized, compared to EL. Overall, this results in more natural speech capable of providing a QoL comparable to organ-preserved patients [25]. The limiting factor for the diffusion of this rehabilitation technique is that it requires a voice prosthesis acting as a unidirectional valve to impede bolus and saliva from entering the airway. These VPs have a variable lifespan. Historical data suggest an average lifetime of 4 to 6 months, but these data have not been revisited in a contemporary practice where TLP is often performed as a salvage procedure after radiation failure. A history of previous radiotherapy significantly affects the device lifetime, resulting in a drop in duration of fewer than two months [26]. Moreover, VPs are required for life, carrying significant costs, especially for patients living in countries where the health care system or health insurance is not provided freely. Therefore, new solutions have been explored to provide the same advantages as TEP without requiring a disposable device. In this context, the J-flap technique, first described by Tsao in 2020 [14], exploits a similar mechanism to TEP, i.e., the tracheoesophageal fistula, without the need for a voice prosthesis as the J tube conformation acts as a valve itself. This technique has already been proved to have satisfactory vocal results [11], but comparisons with the gold standard voice restoration method TEP have never been conducted. This study was designed to comprehensively assess various aspects of the voice rehabilitation outcome: the patients’ subjective perception of their voice (VHI) and QoL (UW-QoL), the professional assessment by speech pathologists (GIRBAS, speech intelligibility and speech accuracy) and the objective evaluation of phonatory and acoustic parameters.

What emerged from the speech pathologists’ assessment was that TEP voices were slightly but significantly less dysphonic compared to J-flap voices, which might be penalized by a more rough, breathy and strained sound. In this study, the tumor site showed significant differences between the two groups, with a prevalence of the larynx in the TEP group, and the pharynx in the J-flap group (*p* < 0.001). Moreover, there was a statistically significant (*p* < 0.001) difference in the radiotherapy administration both in the preoperative and postoperative settings. The different distributions in the site involved and radiotherapy delivered might partially explain the poorer performance of the J-flap cohort. J-flap patients underwent a wider pharyngeal mucosa sacrifice, with a resultant larger area to reconstruct with the stiffer skin of the ALT. Furthermore, the post-actinic changes might affect the residual mucosal vibration.

On the other hand, phonatory and acoustic performances for the two techniques were comparable with MPTs, in line with others reported in the literature for TEP rehabilitation [27]. F0 turned out to be closer to healthy males’ values for J-flap patients, although this difference did not reach statistical significance due to the large variability within both groups. The perturbation values (jitter and shimmer) were similar in the two groups. In contrast, noise values were shown to be pathological in both groups, as expected. Moreover, the average HNR in the two groups was similar to that reported by Stajner-Katusic et al. [28] for TEP-rehabilitated patients (3.41 vs. 3.24), while the average NHR for the TEP group was comparable with that measured by van As et al. [27] for TEP-rehabilitated patients (0.65 vs. 0.50).

According to patients’ perception, TEP voices sounded less impaired than J-flap voices in every domain of the VHI, outlining a more enthusiastic acceptance of the rehabilitated voice for the TEP cohort. Patients’ satisfaction was also reflected in the QoL scores, which were higher in each of the 12 items of the UW-QoL for the TEP group. Consequently, the global composite score revealed a significantly better QoL for TEP patients. Nevertheless, as the UW-QoL considers many aspects of social and emotional life, these results are hardly attributable only to the voice rehabilitation technique; as a matter of fact, they might also be influenced by the different oncological histories due to the severity of disease and the need for salvage treatment after CCRT due to a recurrent and residual tumor.

In the context of salvage surgery, free flap transfer has been demonstrated to represent a valid option even in situations where primary closure of the pharynx is otherwise possible [29]; it may aid with healing and decrease the risk of wound complications and pharyngo-cutaneous fistulas [30]. This evidence has encouraged the Taiwanese department to extend indications for the J-flap method to patients undergoing salvage TL who did not require free flap reconstruction before, combining the benefits mentioned above with the synchronous restoration of the speech function. Further study to compare the outcomes between patients with hypopharyngeal cancer undergoing J-flap reconstruction and patients with laryngeal cancer is worth being performed in the future.

Finally, a more exhaustive comparison between the two techniques should be carried out to better evaluate them, specifically in patients requiring a flap reconstruction. TEP is currently the gold standard for voice restoration after TPL and TL: nevertheless, the necessity of continuous replacement of voice prostheses prevents this technique from being feasible, not only for low-income countries but also for every patient that cannot afford it due to health care system regulations or health insurance policies. Moreover, even when TEP is economically viable, it is not indicated for patients living in isolation or far from the hospital, as this technique bounds them to seriated hospital visits for the rest of their lives: these patients were proven not to be satisfied, as with the others, and often stated that they would not choose this type of voice restoration again [31]. J-flap rehabilitation can overcome these limitations and might find its role for these categories of patients. Nevertheless, the J-flap technique can only reasonably be performed primarily when TL or TLP is required but cannot be advocated subsequent to larynx ablative surgery if the patient changes their mind. In this respect, TEP remains the technique with more indications and more flexibility.

There are several limitations of this study. First, a small number of patients were included in this study, which may be related to the multimodality therapy. Larger cohorts are necessary to generalize the characteristics of each technique in the future. Second, there was only one experienced speech pathologist for the speech intelligibility test, which may result in subjectivity in the evaluation. Nevertheless, the NTID rating scale and speech accuracy were proven to have good intra- and inter-rater reliability and validity in previous studies [15,16].

## 5. Conclusions

According to this study, the TEP and J-flap methods share similar objective phonatory and acoustic outcomes; however, in this study, the voice produced by the latter technique was considered more impaired by the speech pathologists and, ultimately, by the patients themselves. Nevertheless, these differences in the subjective evaluation were not marked, and, in our view, these two voice restoration techniques possess the characteristics to complement each other in actual clinical practice.

## Figures and Tables

**Table 1:** Patients’ demographics.

Variables	Categories	Total		J-Flap		TEP		*p*-Value
		Mean	SD	Mean	SD	Mean	SD	
Age		60.1	8.6	58.33	9.39	61.7	7.71	0.2
Height		1.69	0.06	1.65	0.05	1.72	0.04	<0.001
Weight		67.65	9.72	62.82	8.63	72	8.68	0.002
BMI		23.53	2.69	22.96	2.82	24.05	2.53	0.22
		No.	%	No.	%	No.	%	
Sex	Male	38	100	18	100	20	100	
	Female	0	0	0	0	0	0	
Tumor Site	Larynx	22	57.9	4	22.2	18	90	<0.001
	Pharynx	16	42.1	14	77.8	2	10	
Tumor	Primary	16	42.1	4	22.2	12	60	0.019
	Recurrence	22	57.9	14	77.8	8	40	
Radiotherapy	Preoperative	19	50	15	83.3	4	20	<0.001
	Postoperative	18	47.4	3	16.7	15	75	

**Table 2:** Speech pathologist evaluation.

Speech Pathologist Evaluation	J-Flap Group	TEP Group	*p*-Value
Speech intelligibility			
NTID scale	3.50 ± 0.63	4.25 ± 1.11	**0.005**
GIRBAS			
Voice quality Globally	2.446 ± 0.85	1.95 ± 0.82	**0.04**
Voice quality Instability	0.61 ± 0.97	0.80 ± 1.00	0.43
Voice quality Roughness	2.89 ± 0.47	1.60 ± 0.68	**<0.001**
Voice quality Breathiness	0.72 ± 1.22	0.00 ± 0.00	**0.01**
Voice quality Asthenia	0.11 ± 0.32	0.05 ± 0.22	0.49
Voice quality Strain	2.44 ± 0.92	1.55 ± 1.05	**0.009**
Speech accuracy			
Vowel correct rate (%)	96.66 ± 6.51	93.54 ± 17.19	0.29
Consonant correct rate (%)	65.77 ± 11.02	86.02 ± 20.79	**<0.001**
Word correct rate (%)	69.27 ± 11.52	77.56 ± 22.23	0.185

Results are reported as the mean with the standard deviation. National Technical Institute for the Deaf (NTID). Statistically significant results are reported in bold.

**Table 3:** Phonatory performances and acoustic voice analysis.

Acoustic and Phonatory Parameters	J-Flap Group	TEP Group	*p*-Value
Phonatory performances			
Maximum phonation time (s)	12.66 ± 7.36	11.26 ± 5.90	0.53
S/Z ratio	1.06 ± 0.78	1.24 ± 0.35	0.43
Acoustic voice analysis			
Fundamental frequency (Hz)	183.15 ± 114.21	270.20 ± 183.80	0.12
Jitter (%)	9.66 ± 6.99	9.78 ± 7.38	0.96
Shimmer (dB)	1.90 ± 0.96	1.66 ± 0.72	0.39
Harmonic-to-noise ratio	2.13 ± 1.11	1.88 ± 0.91	0.50

**Table 4:** Voice handicap index.

Voice Handicap Index	J-Flap Group	TEP Group	*p*-Value
Physical score	19.78 ± 8.57	5.42 ± 3.87	**<0.001**
Functional score	19.44 ± 10.25	9.11 ± 6.07	**0.0001**
Emotional score	13.33 ± 9.74	3.79 ± 4.46	**<0.001**
VHI total score	52.56 ± 26.78	18.32 ± 11.62	**<0.001**

Results are reported as the mean with the standard deviation. Statistically significant results are reported in bold.

**Table 5:** Quality of life evaluation.

University of Washington Quality of Life (QoL) Questionnaire	J-Flap Group	TEP Group	*p*-Value
Domain			
Physical function subdomain	68.19 ± 21.65	88.33 ± 9.46	**0.009**
Social-emotional function subdomain	76.67 ± 19.14	87.06 ± 7.65	0.09
QoL composite	72.43 ± 18.30	87.69 ± 6.42	**0.016**

Results are reported as the mean with the standard deviation. Statistically significant results are reported in bold.

## Data Availability

The data presented in this study are available on request from the corresponding author.

## Supplementary Materials

The following supporting information can be downloaded at: https://www.mdpi.com/article/10.3390/cancers14030544/s1, Figure S1: Close view of tracheostoma (TEP); Figure S2: Close view of tracheostoma (J-flap); Video S1: The display of speaking function (TEP); Video S2: The display of speaking function (J-flap).
