# Supplementary figures and images for: Comprehensive Evaluation of Vocal Outcomes and Quality of Life after Total Laryngectomy and Voice Restoration with J-Flap and Tracheoesophageal Puncture
Source: Cancers (Basel). 2022 Jan 21;14(3):544. doi: 10.3390/cancers14030544 (PMC8833548; doi:10.3390/cancers14030544)

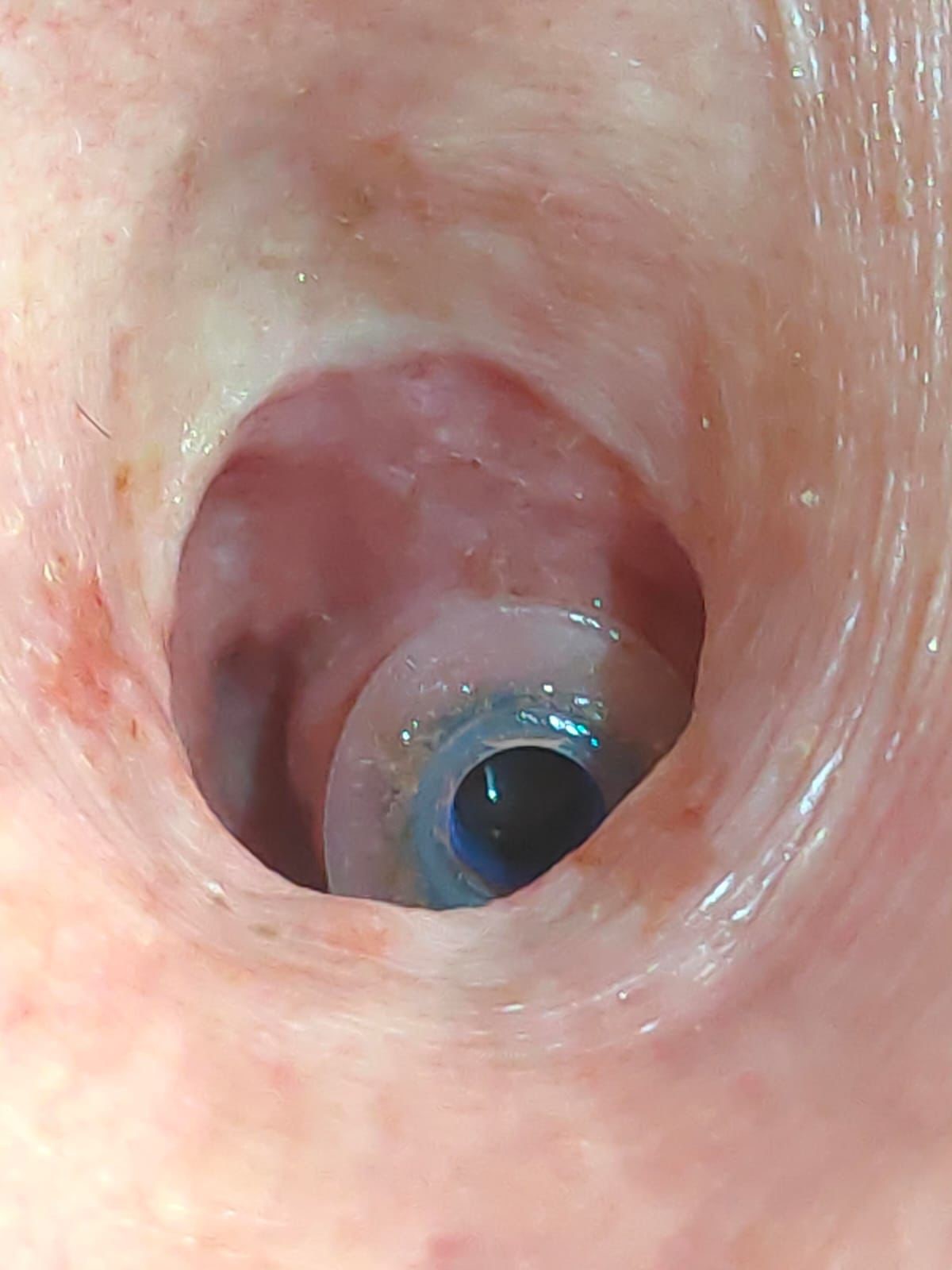

Supplement: Supplementary file 1 [file cancers-14-00544-s001.zip › cancers-1526062 - supp to xml/Figure S1.jpg]

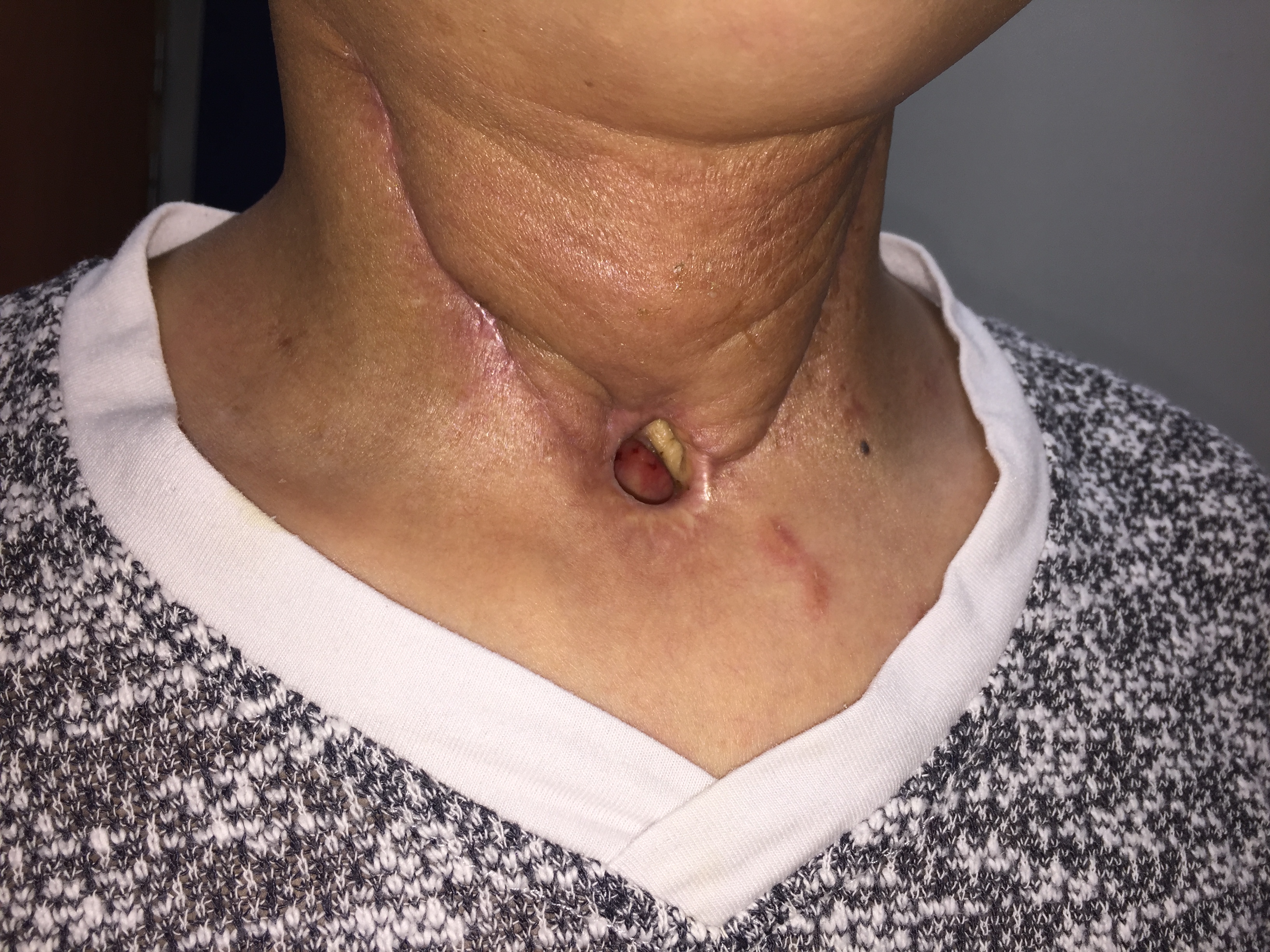

Supplement: Supplementary file 1 [file cancers-14-00544-s001.zip › cancers-1526062 - supp to xml/Figure S2.JPG]
